# The Xanthine Oxidase Inhibitor Febuxostat Suppresses the Progression of IgA Nephropathy, Possibly via Its Anti-Inflammatory and Anti-Fibrotic Effects in the gddY Mouse Model

**DOI:** 10.3390/ijms19123967

**Published:** 2018-12-10

**Authors:** Masa-Ki Inoue, Takeshi Yamamotoya, Yusuke Nakatsu, Koji Ueda, Yuki Inoue, Yasuka Matsunaga, Hideyuki Sakoda, Midori Fujishiro, Hiraku Ono, Kenichi Morii, Kensuke Sasaki, Takao Masaki, Yusuke Suzuki, Tomoichiro Asano, Akifumi Kushiyama

**Affiliations:** 1 Department of Medical Science, Graduate School of Medicine, Hiroshima University, 1-2-3 Kasumi, Minami-ku, Hiroshima City, Hiroshima 734-8551, Japan; b131831@hiroshima-u.ac.jp (M.-K.I.); ymmty@hiroshima-u.ac.jp (T.Y.); nakatsu@hiroshima-u.ac.jp (Y.N.); urouedakouji@yahoo.co.jp (K.U.); iyuki_ym@yahoo.co.jp (Y.I.); 2 Center for Translational Research in Infection & Inflammation, School of Medicine, Tulane University, 6823 St. Charles Avenue, New Orleans, LA 70118, USA; ymatsunaga@tulane.edu; 3 Division of Neurology, Respirology, Endocrinology and Metabolism, Department of Internal Medicine, Faculty of Medicine, University of Miyazaki, 5200 Kihara, Kiyotake, Miyazaki 889-1692, Japan; hideyuki_sakoda@med.miyazaki-u.ac.jp; 4 Division of Diabetes and Metabolic Diseases, Nihon University School of Medicine, Itabashi, Tokyo 173-8610, Japan; fujishiro.midori@nihon-u.ac.jp; 5 Department of Clinical Cell Biology, Graduate School of Medicine, Chiba University, 1-8-1 Inohana, Chuo-ku, Chiba City, Chiba 260-8670, Japan; hono@chiba-u.jp; 6 Department of Nephrology, Hiroshima University Hospital, 1-2-3 Kasumi, Minami-ku, Hiroshima City, Hiroshima 734-8551, Japan; kenichi_morii@yahoo.co.jp (K.M.); sasakikuma@gmail.com (K.S.); masakit@hiroshima-u.ac.jp (T.M.); 7 Division of Nephrology, Department of Internal Medicine, Juntendo University Faculty of Medicine, Hongo 2-1-1, Bunkyo-ku, Tokyo 113-8421, Japan; yusuke@juntendo.ac.jp; 8 Division of Diabetes and Metabolism, The Institute for Adult Diseases, Asahi Life Foundation, Chuo-ku, Tokyo 103-0002, Japan

**Keywords:** IgA nephropathy, xanthine oxidase inhibitor, Febuxostat, Inflammation, Fibrosis

## Abstract

Recent clinical studies have demonstrated the protective effect of xanthine oxidase (XO) inhibitors against chronic kidney diseases, although the underlying molecular mechanisms remain unclear. However, to date, neither clinical nor basic research has been carried out to elucidate the efficacy of XO inhibitor administration for IgA nephropathy. We thus investigated whether febuxostat, an XO inhibitor, exerts a protective effect against the development of IgA nephropathy, using gddY mice as an IgA nephropathy rodent model. Eight-week-old gddY mice were provided drinking water with (15 μg/mL) or without febuxostat for nine weeks and then subjected to experimentation. Elevated serum creatinine and degrees of glomerular sclerosis and fibrosis, judged by microscopic observations, were significantly milder in the febuxostat-treated than in the untreated gddY mice, while body weights and serum IgA concentrations did not differ between the two groups. In addition, elevated mRNA levels of inflammatory cytokines such as TNFα, MCP-1, IL-1β, and IL-6, collagen isoforms and chemokines in the gddY mouse kidneys were clearly normalized by the administration of febuxostat. These data suggest a protective effect of XO inhibitors against the development of IgA nephropathy, possibly via suppression of inflammation and its resultant fibrotic changes, without affecting the serum IgA concentration.

## 1. Introduction

The search for methods of preventing chronic kidney disease (CKD) progression is still an important and ongoing challenge [1]. Usually, lifestyle and dietary modifications aimed at reducing sodium intake as well as renin-angiotensin-aldosterone system blockers are recommended [2,3,4]. On the other hand, several recent clinical studies have suggested that xanthine oxidase inhibitors (XOi), including febuxostat, beneficially inhibit CKD progression [5]. Experimentally, febuxostat also exerts direct renal benefits, independently of its uric acid (UA) lowering effect [6,7,8,9]. Although reduction of oxidative stress by XO inhibition might be involved in the underlying mechanism, clear evidence has not as yet been obtained. At present, the diseases impacted by the preventive effects of XOi on renal failure progression, which appear to include diabetes mellitus, glomerular sclerosis, chronic glomerulonephritis, and various autoimmune disorders, remain to be fully clarified.

IgA nephropathy is a kidney disease characterized by deposition of immunoglobulin A (IgA) in the glomeruli. This results in local chronic inflammation, leading to glomerular injury and impairment of renal functions such as filtration [10]. The courses of IgA nephropathy vary greatly among patients, although approximately 30% will have end-stage renal disease within 20 years of the initial diagnosis [11]. At present, there is no definitive cure for IgA nephropathy, with blood pressure control and/or the usage of anti-inflammatory agents such as steroids only slowing its progression [12,13,14,15].

We investigated the effects of febuxostat, one of the widely used XOis, on the development of IgA nephropathy, using gddY mice, a rodent model of spontaneous IgA nephropathy [16]. Unlike HIGA mice showing only mild proteinuria without hematuria, gddY mice exhibit an early onset of glomerular IgA deposition and clinicopathological aberrations other than hematuria, features resembling those of human IgA nephropathy [17]. To date, neither clinical nor basic research has been carried out regarding the efficacy of XOi as a treatment for IgA nephropathy. Thus, to our knowledge, this is the first study to demonstrate the benefits of XOis for treating IgA nephropathy. In particular, febuxostat treatment markedly suppressed inflammation and the resultant collagen deposition in the kidneys of gddY mice. These results suggest the potential of febuxostat as a treatment for human IgA nephropathy.

## 2. Results

### 2.1. Xanthine Oxidase Inhibitor Febuxostat Prevents Progression of IgA Nephropathy

**Figure 1:**
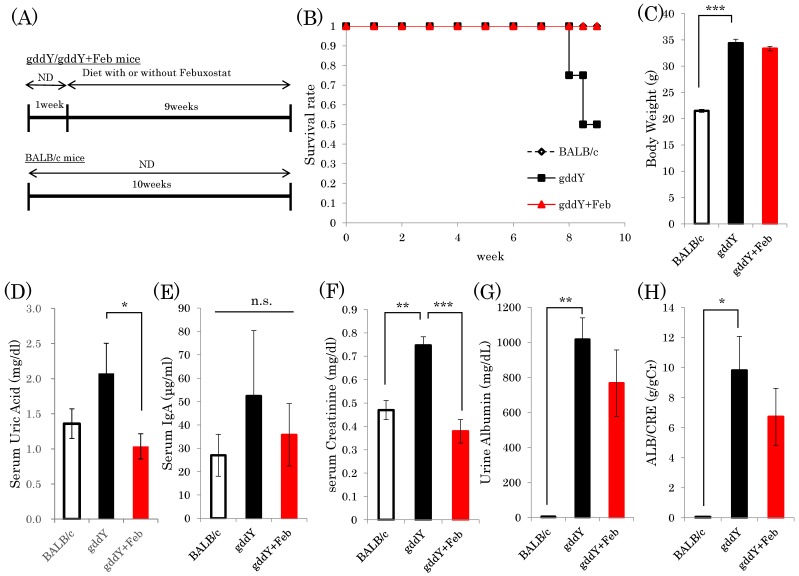
Xanthine oxidase inhibitor febuxostat prevents progression of IgA nephropathy. (**A**) Mice were divided into three groups: gddY mice with or without febuxostat treatment and BALB/c control mice (*n* = 8). After 9 weeks, the mice were sacrificed and kidneys harvested. (**B**) Survival rates of mice at 9 weeks after the start of experiments are shown. (**C**) At 9 weeks, body weights were measured. (**D**–**F**) Serum UA, IgA, and creatinine contents were determined, respectively. (**G**,**H**) Urine albumin levels were measured and the ALB/CRE ratio was calculated. *: *p* < 0.05, **: *p* < 0.01, ***: *p* < 0.001. n.s.: not significant.

Sixteen nine-week-old female gddY mice were divided into two groups and were given water either with (15 μg/mL) or without febuxostat (Figure 1A). At nine weeks after the initiation of febuxostat, four of the eight untreated gddY mice had died, while all eight gddY mice receiving febuxostat in their drinking water survived (Figure 1B). Although a previous report indicated gddY mice to have a higher death rate than normal mice [18], the death rate of untreated gddY mice in the present study exceeded our expectations. We speculate that these deaths were due to renal failure but have no proof. Fortunately, there were four untreated gddY mice that survived and appeared to be as healthy as the febuxostat-treated gddY mice and the normal control mice, judging from their activities and other parameters. Thus, the following experiments were performed by analyzing three groups, i.e., the four untreated gddY, eight febuxostat-treated gddY and eight normal BALB/c mice. While the gddY mice weighed significantly more than BALB/c mice, febuxostat treatment did not affect the weights of gddY mice (Figure 1C). Serum uric acid concentration was significantly lowered in febuxostat-treated gddY mice compared to untreated gddY mice (Figure 1D). Serum IgA levels showed large variations among individual mice, but differences among the three groups were not significant (Figure 1E).

Serum creatinine content was significantly elevated in the gddY mice, but normalized in response to febuxostat treatment, reaching a value similar to that of normal mice (Figure 1F). In addition, both urinary albumin amount and the albumin/creatinine ratio in gddY mice were significantly increased, as compared to those in control mice. Treatment with febuxostat blunted these elevations, though not to a statistically significant degree (Figure 1G,H). These results indicate febuxostat administration to exert reno-protective effects in IgA model mice.

### 2.2. Febuxostat Suppresses Nephropathy Development without Significantly Affecting Glomerular IgA Deposition

**Figure 2:**
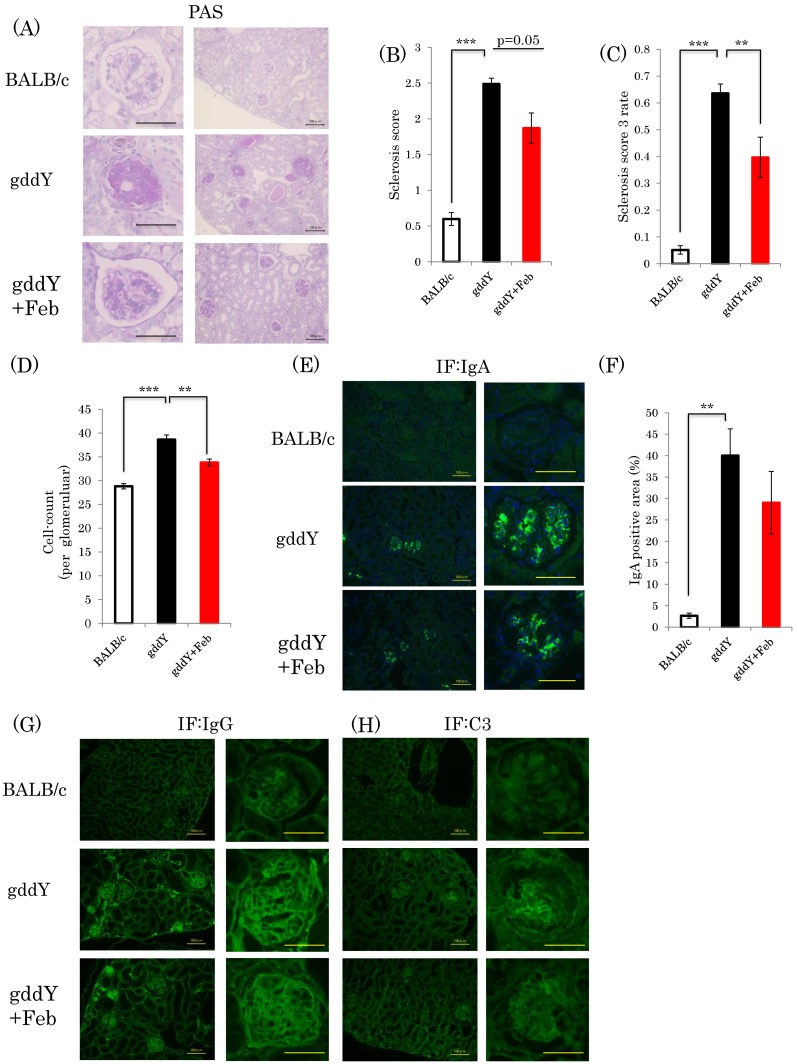
Febuxostat suppresses the development of nephropathy with no significant effects on glomerular IgA deposition. (**A**) PAS staining of kidney fractions. scale bar = 50 μm (left panel), 100 μm (right panel); (**B**) Sclerosis scores were calculated. (**C**) Rate of sclerosis score 3 in glomerular specimens; (**D**) Glomerular cell count; (**E**) Immunostaining of renal sections with anti-IgA antibody. scale bar = 100 μm (left panel), 50 μm (right panel); (**F**) IgA positive areas in mesangial cells were measured; (**G,H**) Immunostaining of renal sections with anti-IgG antibody or anti-C3 antibody, scale bar = 100 μm (left panel), 50 μm (right panel). (BALB/c control mice: *n* = 8, untreated gddY mice: *n* = 4, febuxostat treated gddY mice: *n* = 8) **: *p* < 0.01, ***: *p* < 0.001.

Glomerular sclerosis was previously shown to be involved in the development of chronic renal failure due to IgA nephropathy [19]. Glomerular sclerosis scores based on periodic acid Schiff (PAS) staining were significantly elevated in gddY mice as compared with the normal controls (Figure 2A). Based on PAS staining and calculated sclerosis scores, the development of glomerular sclerosis appeared to be milder in the febuxostat-treated gddY than in the untreated gddY mice, but this difference did not reach statistical significance (*p* = 0.05) (Figure 2B). However, the proportion of glomeruli in which the sclerotic region was at least 50%, corresponding to a sclerosis score of 3, was significantly lower in the febuxostat-treated gddY than in the untreated gddY mice (Figure 2C). The mesangial cell proliferation is another feature of IgA nephropathy. The glomerular cell count results showed numbers of cells to be significantly increased in the gddY mice, but febuxostat treatment normalized these counts (Figure 2D). Marked IgA, IgG, and C3 depositions were observed in glomeruli of gddY mice, but febuxostat administration had no apparent effects on IgA, IgG, and C3 accumulations (Figure 2E–H).

### 2.3. Febuxostat Suppresses Inflammation—Related Gene Expressions in the Kidneys of gddY Mice

**Figure 3:**
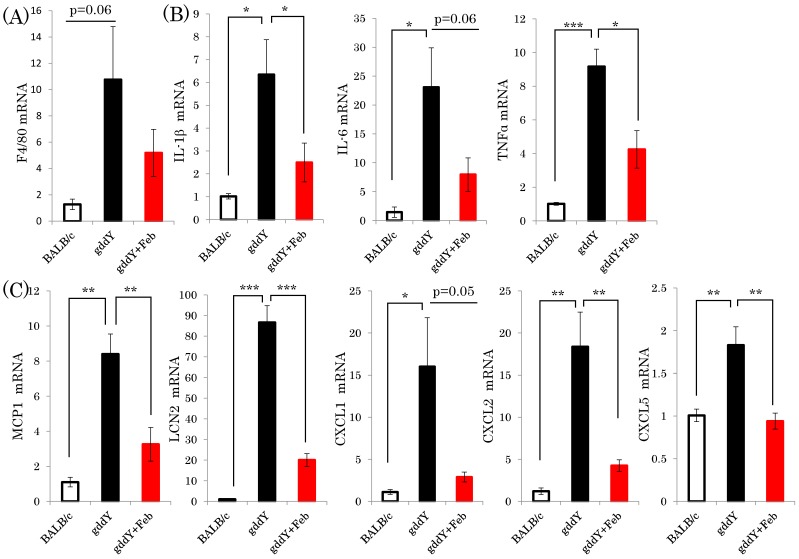
Febuxostat suppresses the expressions of both inflammatory cytokines and chemokines in the kidneys of gddY mice. (**A**–**C**) Relative mRNA levels of macrophage markers, cytokines, and chemokines in the kidneys were determined employing real time PCR (BALB/c control mice: *n* = 8, untreated gddY mice: *n* = 4, febuxostat treated gddY mice: *n* = 8) *: *p* < 0.05, **: *p* < 0.01, ***: *p* < 0.001.

During the process of renal failure development in IgA nephropathy, inflammatory responses and subsequent fibrotic changes triggered by glomerular IgA deposition play critical roles [20]. Thus, we investigated how febuxostat affects the expressions of various inflammation-related genes in the gddY murine model. Expressions of macrophage markers F4/F80, which were elevated in the gddY mouse kidneys, were normalized by febuxostat treatment, although these differences did not reach statistical significance (Figure 3A). Notably, expressions of both inflammatory cytokines (IL-1β, IL-6, TNFα, and MCP-1) and chemokines (LCN2, CXCL1, CXCL2, and CXCL5) in the kidneys were markedly elevated in the untreated gddY mice as compared with the normal controls, but these elevations were blunted by febuxostat administration (Figure 3B,C).

### 2.4. Febuxostat Ameliorates Kidney Fibrosis

**Figure 4:**
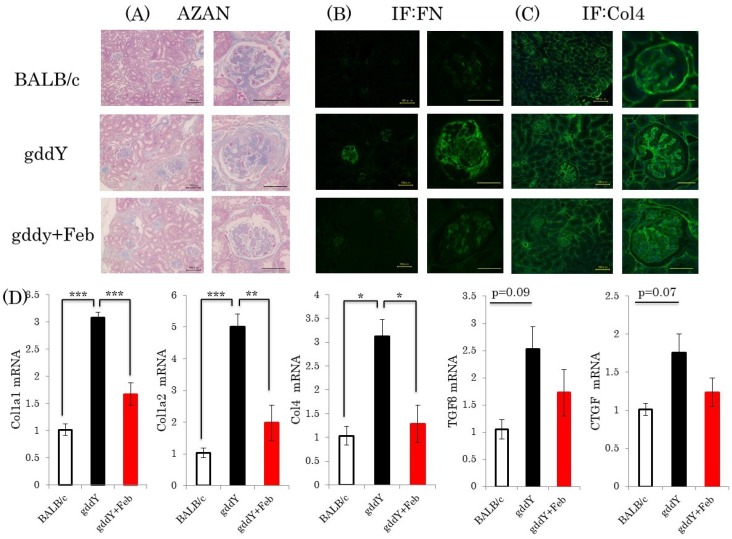
Febuxostat ameliorates kidney fibrosis (**A**) AZAN staining of kidney fractions, scale bar = 100 μm (left panel), 50 μm (right panel); (**B**,**C**) Immunostaining of renal sections with anti-FN or Col4 antibody. Representative images are shown, scale bar = 100 μm (left panel), 50 μm (right panel); (**D**) Relative mRNA levels of fibrotic markers in the kidneys were measured. (BALB/c control mice: *n* = 8, untreated gddY mice: *n* = 4, febuxostat treated gddY mice: *n* = 8) *: *p* < 0.05, **: *p* < 0.01, ***: *p* < 0.001.

Fibrotic change is one of the major characteristics of end-stage renal disease [21]. Thus, we investigated the effects of febuxostat on the expressions of genes related to the fibrotic process. We also performed microscopic observations by AZAN staining and immunochemical staining with anti-fibronectin and anti-collagen 4 antibodies. Whereas remarkable fibrosis was observed in the kidneys of non-treated gddY mice, febuxostat significantly diminished fibrotic areas in the kidneys of mice with IgA nephropathy (Figure 4A–C). Consistent with these results, expressions of fibrotic markers, such as collagen 1a1, collagen 1a2, collagen 4a1, CTGF, and TGFβ, which were elevated in the gddY mouse kidneys, were normalized by febuxostat treatment (Figure 4D).

### 2.5. Febuxostat Prevents Progression of IgA Nephropathy by Inhibiting Xanthine Oxidase

**Figure 5:**
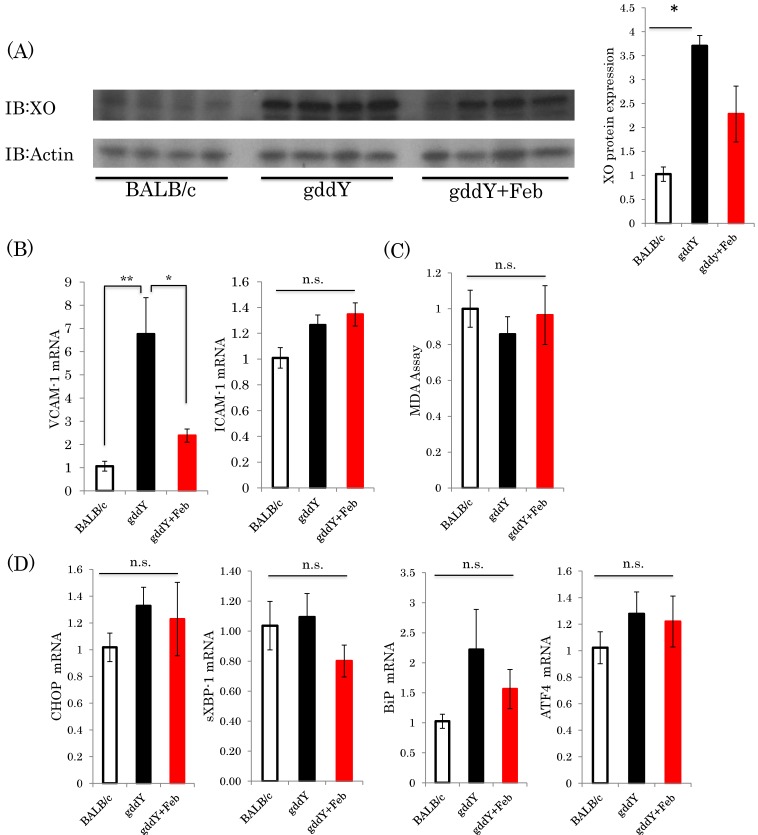
Febuxostat reduced the xanthine oxidase protein expression level but had no effects on oxidative or ER stress parameters. (**A**) XO and Actin protein expressions were examined by immunoblotting, and four representative blots from each group are shown (left panel); quantitation data are presented as a bar graph (right panel). (BALB/c control mice: *n* = 8, untreated gddY mice: *n* = 4, febuxostat treated gddY mice: *n* = 8); (**B**) Relative mRNA levels of VCAM-1 and ICAM-1 in the kidneys; (**C**) TBARS assay; (**D**) Relative mRNA levels of CHOP, sXBP1, BiP, and ATF4 in the kidneys. (BALB/c control mice: *n* = 8, untreated gddY mice: *n* = 4, febuxostat treated gddY mice: *n* = 8). *: *p* < 0.05, **: *p* < 0.01, n.s.: not significant.

To identify the mechanism(s) by which febuxostat suppresses IgA nephropathy, we performed several assays. Interestingly, xanthine oxidase protein expression was significantly increased in the gddY mice as compared with the control group. While febuxostat treatment blunted this increase in gddY mice, the reduction was not statistically significant (Figure 5A). Expressions of endothelial damage markers [22] (such as VCAM-1) in the kidneys were markedly elevated in the untreated gddY mice as compared with the normal controls, but these elevations were blunted by febuxostat administration (Figure 5B).On the other hand, TBARS assay, which quantifies the amount of malondialdehyde (MDA), the product of lipid peroxidation, showed no apparent difference between the groups in terms of the oxidative stress levels in the kidney (Figure 5C). The mRNA levels of several endoplasmic reticulum stress markers, such as CHOP, spliced XBP1 (sXBP1), BiP, and ATF4, were also investigated, and showed no significant changes (Figure 5D).

## 3. Discussion

Herein, we demonstrated for the first time that the XOi febuxostat exerts potent anti-inflammatory actions, i.e., the suppression of inflammatory cytokine and chemokine expressions in the kidney which were markedly elevated in the IgA nephropathy rodent model gddY mice. The reno-protective effect produced by febuxostat was evidenced by suppression of glomerular sclerosis and creatinine elevation. In contrast, febuxostat treatment exhibited no apparent effects on the elevated serum IgA level, increased renal glomerular IgA deposition, or increased urinary albumin excretion in gddY mouse kidneys. No obvious effects of febuxostat could be found in either oxidative stress or endoplasmic reticulum stress in this model.

The “multi-hit” hypothesis was recently proposed to explain the pathogenesis of IgA nephropathy progression, which includes at least four steps [23]. At the fourth so-called hit, mesangial deposition of IgA immune complexes takes place, in which IgA abnormalities and generation of the resultant autoantibodies appear to play a critical role. Glomerular injury caused by immunocomplex deposition subsequently leads to the secretion of inflammatory cytokines [24,25,26]. Expressions of numerous cytokines, chemokines, and growth factors from mesangial and immune cells, within the kidneys, are considered to contribute to mesangial cell proliferation, extracellular matrix overexpression, glomerulosclerosis, and fibrosis. We previously demonstrated XO to activate inflammatory cells such as macrophages in blood vessels [27] and hepatic [28] systems. XOis suppressed inflammation and oxidative stress [29] in macrophages, and fibrosis in atherosclerosis and a non-alcoholic steatohepatitis model.

Taking the aforementioned theoretical process of IgA nephropathy development and progression, as well as our data, into consideration, the mechanism by which febuxostat protects against IgA nephropathy is suggested to involve its anti-inflammatory properties. We found the local XO protein level to be upregulated in the kidneys of gddY mice. Expression of XO is known to be induced by inflammation, hypoxia [30], and the renin-angiotensin system [31]. XO physiologically defends against renal interstitial fibrosis [32] by preventing or reducing these inductions, while excessive XO plays crucial roles in glomerular injury via inflammatory processes and fibrosis in the gddY model. Since XOis including febuxostat have already been clinically applied and their safety has been relatively well established [33], treating IgA nephropathy by XOi in a clinical trial might be worth considering.

The observations that albumin secretion is not reduced but ameliorated serum creatinine levels are speculated to reflect the protective effect of febuxostat against progression to late-stage nephropathy. There are reported variations of the effects of XOis on renal function. In the diabetic db/db mouse model, XOis such as allopurinol and topiroxostat reduced urinary albumin excretion [34,35]. In patients with CKD, treatment with the XOi topiroxostat resulted in significant reductions in serum UA and urinary protein excretion but not in the estimated glomerular filtration rate (eGFR) [36]. The eGFR was maintained in CKD patients treated with febuxostat, and the reno-protective effects were reportedly masked by vascular risk factors such as diabetes and hypertension [37]. Therefore, CKD pathology and the type of medication administered appear to be critical for achieving the reno-protective effects.

There are two possible mechanisms linking UA metabolism and inflammation; one is inflammation activated by UA crystallization and the other involves generation of superoxide free radicals during UA synthesis [30,38]. Since mice have far lower serum UA concentrations than humans, it is unlikely that UA crystals are crucial in murine models. In contrast, generation of free radicals via XO activity is basically independent of the UA concentration. However, we detected no differences in oxidative stress levels in the whole kidney, between either gddY and normal mice or febuxostat-treated and untreated gddY mice. We speculate that the generation of oxidative stress was limited to a highly localized area and that changes in the whole kidney might thus have been difficult to detect. Thus, it is debatable to conclude that the reno-protective effect of febuxostat is not due to the reduction of free radical generation, and further studies are necessary to clarify this issue.

## 4. Materials and Methods

### 4.1. Animals

Eight-week-old female gddY mice maintained in Juntendo University and control BALB/c mice purchased from CLEA Japan were used for the experiments. After 1 week of acclimation, gddY mice were divided into two groups, without or with febuxostat mixed into their drinking water (15 μg/mL). All groups were fed normal diet and had free access to water and food. After 9 weeks of treatment, all mice were killed, and the kidneys and blood samples were collected for further analysis. Blood samples were incubated on ice for 30 min and then centrifuged at 1500 rpm for 30 min at 4 °C to separate serum. All samples were preserved at −80 °C. All animals were handled in accordance with the guidelines for the care and use of experimental animals published by Hiroshima University.

### 4.2. Reagents

Febuxostat was generously provided by Teijin Pharma Ltd. (Tokyo, Japan). Antibodies were purchased from Abcam (Cambridge, MA, USA) (Fibronectin-ab23750, Collagen4-ab6586, C3-ab11862, IgG-ab190475), Santa Cruz (Dallas, TX, USA) (actin sc-47778 F1417, XO sc-20991 A0705), and BETHYL (Montgomery, TX, USA) (IgA A90-104A).

### 4.3. Metabolic Analysis

Mouse urine was collected for 24 h employing metabolic cages and preserved at −80 °C. Urine albumin, serum IgA, and urine and serum creatinine were quantified using an albumin ELISA Kit (WAKO, Osaka, Japan), mouse IgA ELISA Kit (Crystal Chem, IL USA), and creatinine ELISA Kit (WAKO, Osaka, Japan), respectively. All assays were performed according to the manufacturers’ protocols. Serum UA concentrations were determined with a UA assay kit (Cayman Chemical, MI), according to the manufacturer’s instructions.

### 4.4. Histological Study

Paraffin-embedded kidney sections were subjected to AZAN and PAS staining to identify fibrotic change and glomerular sclerosis, respectively. For IgA, C3, IgG, collagen 4, and fibronectin immunostaining, deparaffinized sections were treated as follows. Samples were incubated with 0.1% Triton solution for 5 min and 100 μg/L Proteinase K for 30 min to achieve antigen activation. After being washed, the sections were incubated with the corresponding primary antibodies (1/200) at 4 °C overnight. Next, the sections were incubated with FITC-conjugated secondary antibodies for 60 min at room temperature. After being washed with PBS, slides were embedded with DAPI. The samples were analyzed using a microscope (HS All-in-one Fluorescence Microscope BZ9000E, KEYENCE, Osaka, Japan) and images were saved and stored in the JPEG format.

For the quantitative analysis of glomerular sclerosis, more than 30 glomeruli/cross-section were observed, and scores were assigned based on the method previously reported by Okazaki et al. as follows: 0 points, no glomerular sclerosis; 1 point mild glomerular sclerosis (approximately 25%); 2 points, moderate glomerular sclerosis (<50%); and 3 points, severe glomerular sclerosis (>50%). Sclerosis scores were calculated as follows: [∑(each score × number of glomeruli)]/number of glomeruli [17,21].

The numbers of cells in 30 glomeruli per mouse were counted to quantify mesangial cell proliferation as previously described [39].

### 4.5. Measurement of Glomerular IgA Positive Area

Immunofluorescence image files were analyzed by Image J (NIH, Bethesda, MD, USA). The color immunofluorescence images were converted to binary data and pixels in black within whole traced (glomerular) areas were counted using the *Analyze Particle* command. We regarded the brightness positive area in the immunofluorescence images as corresponding to a range of pixel values from 51 to 255. To measure the glomerular IgA positive area, the edges of glomeruli were traced. IgA positive areas (Area-IgA, %) were calculated as the glomerular IgA positive area/total glomerular area [40].

### 4.6. MDA Assay

MDA assays were performed employing a TBARS Kit (Cayman, MI, USA). Assays were performed following the manufacturer’s protocol and absorbance was measured at 540 nm.

### 4.7. Western Blotting

Kidneys were homogenized in lysis buffer containing 50 mM Tris-HCl (pH 7.4), 150 mM NaCl, 1 mM ethylenendiaminetetraacetic acid, 1% Triton X-100, 1 mM NaF, 1 mM Na_3_VO_4_, and 1 mM phenylmethylsulfonyl fluoride. The lysates were incubated on ice for 30 min and then centrifuged at 15,000 rpm for 30 min at 4 °C. After adjustment of the protein concentrations, the supernatants were mixed and boiled with sample buffer. Proteins were separated by SDS-PAGE and then transferred to polyvinylidene difluoride membranes. After blocking with 3% skim milk, membranes were incubated with primary antibody (1:2000) overnight at 4 °C The membranes were next washed with PBS three times and then reacted with the secondary antibodies (1:4000) for 1 h at room temperature. After washing, proteins were detected, using Supersignal West Pico Substrate (Thermo Scientific, Waltham, MA, USA) or ImmunoStar LD (Wako).

### 4.8. Real-Time PCR

Total RNA from kidneys was isolated using Sepasol reagent (Nacalai Tesque, Kyoto, Japan). 1 μg of RNA was reverse transcribed using the Verso cDNA Synthesis Kit (Thermo Scientific), according to the kit instructions. Real-time PCR was performed using the CFX96 real-time PCR system (Bio-Rad, Hercules, CA, USA) with KAPA SYBR Green. The primers used are shown in Table 1.

### 4.9. Statistical Analysis

Data were processed using EZR (Saitama Medical Center, Jichi Medical University, Saitama, Japan). Values are presented as means ± S.E.M. Statistical analyses were performed using Student’s unpaired *t*-test for comparing two groups, and one-way ANOVA followed by the post-hoc Tukey’s test for multiple comparisons. A value of *p* < 0.05 was taken to indicate a statistically significant difference.

## 5. Conclusions

This is the first study to raise the possibility that XOi exert a protective effect against the development of IgA nephropathy, in a rodent model. Particularly, renal inflammation and the resultant fibrotic changes were markedly suppressed with febuxostat treatment, which might be the mechanism underlying the reno-protective effect of this XOi. Clinical studies are necessary to determine whether the favorable effects obtained in our rodent model would also occur in human IgA nephropathy patients.

## Figures and Tables

**Table 1:** The list of primer sequences.

Gene	Forward Primer	Reverse Primer
mCHOP	CCACCACACCTGAAGCAGAA	AGGTGAAAGGCAGGGACTCA
mCTGF	CAAAGCAGCTGCAAATACCA	GGCCAAATGTGTCTTCCAGT
mCol1a1	GAGCGGAGAGTACTGGATCG	GCTTCTTTTCCTTGGGGTTC
mCol1a2	CCGTGCTTCTCAGAACATCA	GAGCAGCCATCGACTAGGAC
mCol4a1	GGCTCTGGCTGTGGAAAA	CCAGGTTCTCCAGCATCACC
mIL-1	AGCTGCCACAGCTTCTCCA	TTGACGGACCCCAAAAGATG
mIL-6	CCATCCAGTTGCCTTCTTGG	TGCAAGTGCATCATCGTTGT
mMCP1	AGGTCCCTGTCATGCTTCTG	TCTGGACCCATTCCTTCTTG
mTNFa	GAACTGGCAGAAGAGGCACT	AGGGTCTGGGCCATAGAACT
mTGFb1	TTGCTTCAGCTCCACAGAGA	TGGTTGTAGAGGGCAAGGAC
mLCN2	CCAGTTCGCCATGGTATTTT	TCCTTCAGTTCAGGGGACAG
mCXCL1	CACCCAAACCGAAGTCATAG	AAGCCAGCGTTCACCAGA
mCXCL2	TCCAGAGCTTGAGTGTGACGC	TGGATGATTTTCTGAACCAGGG
mCXCL5	GGTCCACAGTGCCCTACG	GCGAGTGCATTCCGCTTA
mF4/80	TCTGGGGAGCTTACGATGGA	TAGGAATCCCGCAATGATGG
mVCAM	TCTTGGGAGCCTCAACGGTA	TGACAGGCTCCATGGTCAGA
msXBP1	CTGAGTCCGAATCAGGTCCAG	GTCCATGGGAAGATGTTCTGG
mBiP	TTCAGCCAATTATCAGCAAACTC	TTTTCTGATGTATCCTCTTCACC
mATF4	GGGTTCTGTCTTCCACTCCA	AAGCAGCAGAGTCAGGCTTCC
mGAPDH	TGATGGGTGTGAACCACG	GGGCCATCCACAGTCTTCTG
